# Relationships between parents’ academic backgrounds and incomes and building students’ healthy eating habits

**DOI:** 10.7717/peerj.4563

**Published:** 2018-05-03

**Authors:** Kazi Enamul Hoque, Kazi Fardinul Hoque, Revethy A/P Thanabalan

**Affiliations:** 1 Department of Management, Planning and Policy, Faculty of Education, University of Malaya, Kuala Lumpur, Malaysia; 2 Methodist College, Brickfield, Kuala Lumpur, Malaysia

**Keywords:** Parents’ education, Parents’ income, Building students’ healthy eating habit

## Abstract

**Background:**

Building healthy eating habit is essential for all people. School and family are the prime institutions to instill this habit during early age. This study is aimed at understanding the impact of family such as parents’ educations and incomes on building students’ healthy eating habits.

**Methods:**

A survey on building students’ eating habits was conducted among primary school students of grade 4 (11 years) and 5 (12 years) from Kulim district, Malaysia. Data from 318 respondents were analysed. Descriptive statistics were used to find the present scenario of their knowledge, attitude and practices towards their eating habits while one-way ANOVA and independent sample *t*-test were used to find the differences between their practices based on students’ gender, parents’ educations and incomes.

**Results:**

The study finds that the students have a good knowledge of types of healthy food but yet their preferences are towards the unhealthy food. Though the students’ gender and parents’ educations are not found significantly related to students’ knowledge, attitude and practices towards healthy eating habits, parents’ incomes have significant influence on promoting the healthy eating habit.

**Discussion:**

Findings of this study can be useful to guide parents in healthy food choices and suggest them to be models to their children in building healthy eating habits.

## Introduction

Students’ eating habits are influenced by their parents, genetic factors and environment. The genetic factors lie within the family in which obese children normally have obese parents. Adiposity of the parents is heritable (Liewellyn et al., 2013). This attribute is related to the general metabolic rate and also the behavioural genetic of their parents specifically eating style (Van der Horst & Sleddens, 2017; Kral & Rauh, 2010). Nevertheless, it is also influenced by food preferences, social models, television, child-feeding practices and also parental control, eating style, adiposity and gender. There have also been cases of children dieting in order to control weight gain (Goossens et al., 2012). Dietary pattern is an eating habit directly or indirectly modelled by parents (Vollmer & Baietto, 2017) or other close family members to the child that could have either positive or negative effect towards the child. The environment of the child was shown to be the greater contributor in developing their dietary pattern compared to genetic disposition (Weeland et al., 2015). However, depending on the parent dietary habits, children are susceptible to misguided concepts regarding healthy eating. Family members also have large influences on the children. Furthermore, the family lifestyle such as eating out, less healthy home cooking and lack of weekly family recreational activities (Tee, 1999) contribute to forming their eating habit as well.

Other external environment includes TV commercials and food availability and accessibility are also in prime role to shape their eating habits. Most of food advertisements on TV show food of low nutritional quality, high in sugar, salt and saturated fats. Children who are frequently exposed to such advertisements would choose such food compared to children who are not (Halford et al., 2004). Furthermore, bad food choices and unhealthy eating habits are also dependent on the food made available to them at home and at school.

The school body has been recognized as the centre responsible in guiding students to make correct choices (Ludmila, 2017). School environment and policy are also the prime concern as they spend a lot of time there (Kann, Telljohann & Wooley, 2007). The government of Malaysia seems aware of that and has the expectation to ensure each and every student to be at their optimum health conditions. As children of low-income families seem mostly vulnerable to malnutrition diseases, it has previously taken initiative to provide additional nutrition to children from these families such as *RancanganMakananTambahan (RMT)* or the Supplementary Food Programme (SFP) (Hoque et al., 2016) and *Program SusuSekolah (PSS)* or the School Milk Programme which were operated for students from low-income families. In 2011, Healthy Environment Cabinet Committee (JKPHS) Series No. 1 chaired by the Deputy Prime Minister of Malaysia has decided on strengthening healthy school environments through the implementation of efforts towards a healthy diet and avoids availability of food and unhealthy drinks, in line with the implementation of *Program Bersepadu Sekolah Sihat (PBSS)* or the Integrated School Health Programme (ISHP). Hoque et al. (2016) in their study among 400 school children in Selangor State (capital city) in Malaysia also had the findings of the success of ISHP but it recommends the needs of parents’ cooperation for building students’ healthy eating habit to be highly successful. Following this recommendation, the present study conducted by same corresponding author focusing on the influence of parental education and income over children eating habit. Another study also recommended to examine the relationships between parent and family socio-demographic factors such as income, race/ethnicity and eating behaviours during independent eating occasions (Reicks et al., 2015).

In Malaysia, the number of diabetic cases is expected to increase in 2030 to a total of 2.48 million diabetic patients, a whopping 164% increase from 0.94 million in 2000 based on the report by, Mastura et al. (2008). It indicates that if the food habits of today’s children are not changed then most of them are going to be diabetic in 2030. Another study also (Hoque et al., 2016) reported that 6.35% of the children from the age of 9–10 years old in Selangor were obese. They also reported similar percentage of children within the range who were overweight. Although adult obesity brings the onset of diabetes, childhood obesity also brings about diseases upon obese and overweight children. Obesity is a disease as an effect of overeating, unbalanced meals and sedentary lifestyle (Letchhuman et al., 2010). Obesity is strongly associated with diabetes and the rise in the prevalence of diabetes is due to the prevalence of obesity (Ismail et al., 2002).

It shows that initiatives of school and government alone will not be successful if parents are not aware of that. But parents’ awareness depends on their knowledge, education and sometimes income (Drewnowski & Darmon, 2005; Vollmer & Baietto, 2017). A study shows that the fiasco in applying healthy eating habits was due to parents inept in their skills and knowledge to train good eating habits among their children (Birch & Fisher, 1997). Children initial food experience begins from the mother’s womb. They are exposed to different flavours depending on the mother’s diet. The same situation occurs during infancy to breast-fed babies. Formula-fed babies are in the disadvantage in this matter since they are only exposed to single flavour at a time. This first exposure allows the child to be receptive to new flavours thus increasing food acceptance for more variety of food. Thus, parents’ education, skills and income have been the influential factors in their early stages. A Lithunian COSI study investigated the relationship between parental income, education level and children eating habit in which they found the significant impact of parents’ higher education and income on their daily breakfast and fresh fruit consumption (Petrauskienė, Žaltauskė & Albavičiūtė, 2015). Other studies also reported family income factor linked with higher intake of fruit and vegetables among children (Deshmukh-Taskar et al., 2010; Timlin et al., 2008; Duarte et al., 2014). A recent international study among 4752 children of 12 countries (Age 9–11) has found the positive association in some countries and negative association in some other countries between parental education and children’s overweight and physical activity (Muthuri et al., 2016).

Therefore, parents hold the biggest control over current and future of their children’s eating patterns. Eating habits in children are a mix of child feeding practices from parents, innate eating style and gender. Though many published research reported the prevalence of diet-related diseases among school children (Halford et al., 2004; Weeland et al., 2015; Priya, 2012), a relationship between childhood obesity and quality of life and self-esteem (Li & Hooker, 2010), and effectiveness of dietary programs on school children (Sherina & Rozali, 2004; Ruhaya et al., 2012; Hoque et al., 2016), there has not been much study on the relationships between building children’s eating habits and parents’ levels of education and incomes in Malaysia. Moreover, different global studies (Muthuri et al., 2016; Petrauskienė, Žaltauskė & Albavičiūtė, 2015) reported both positive and negative associations between parental education level and income with children eating habit. From this stand point, a study from the Malaysian perspective aiming at finding the relationships between parents’ level of education and income and building students’ healthy eating habit is immensely important.

### Research objectives and hypotheses

To achieve the aim, the following objectives and hypotheses have been formulated:

1. To understand primary school students’ knowledge, attitudes and practices on building healthy eating habits.
2. To find the difference of knowledge, attitude and practices regarding eating habits in terms of students’ gender.
3. To find difference of knowledge, attitude and practices of building healthy eating habits of students in terms of parents’ academic background.
4. To find the difference of knowledge, attitude and practices of students’ healthy eating habits in terms of parents’ income.

**Research Hypotheses**
Ho1: There is no difference of knowledge, attitude and practices regarding healthy eating habit in terms of students’ gender;
Ho2: There is no difference of knowledge, attitude and practices of students’ healthy eating habit in terms of parents’ academic background; and
Ho3: There is no difference of knowledge, attitude and practices of students’ healthy eating habit in terms of parents’ income.

## Material and Methods

### Research design

The research design was developed to address the research objectives and hypotheses of the study which involved the quantitative method. The current research is a cross-sectional study performed among primary schools in Kulim, Kedah Darul Aman in April 2016. Generally, the research method of this study is a modification of the methods used in the report by Central Health Education Unit (2009), Department of Health of Hong Kong. The questionnaires are adapted from the report for each category of respondents and modified to suit the local standard.

### Population and sample

The population of this study consists of all the primary schools students in Kulim district, Malaysia for the academic year 2016. Kulim district is chosen due to urban and sub-urban area as there is another study by the corresponding author (Hoque et al., 2016) in the capital city. As the previous study was conducted in the capital city, this study was intentionally conducted through convenient sampling method in an urban and semi-urban area. There are differences among city and urban students in terms of food consumption and habit (Noraziah & Mohd Azlan, 2012). This study is also included students’ gender, parents’ education and their income as new variables. There are about 50 schools in this district of which only 18 have intended to participate. The sampling of schools is based on their willingness to participate the study. A request letter is sent to all 50 primary schools in the whole of Kulim district (338 km north–west from the capital Kuala Lumpur, Malaysia) for which the list of schools are obtained from the Ministry of Education website. Therefore, the respondents are chosen from 18 schools unlike the study by Central Health Education Unit (2009) in which schools were chosen based on stratified cluster sampling method. The other schools disagree due to their students’ examination preparation and other businesses. But the principals have not clarified what other businesses they have had. In each selected school, a number of students are chosen randomly. Each respondent is given a set of survey questions to be filled depending on their categories.

### Selection of students

For the ease of answering survey questions, only students’ age 10 and 11 are chosen to answer questions regarding their dietary habits. Students younger than 10 years old are not preferred since the questions might be difficult for them. Students aged 12 years old are not selected to allow them to prepare for their upcoming Primary School Achievement Test (*Ujian Pencapaian Sekolah Rendah*). The total number of students for this study is set at 360 students taking 20 students for each school. Altogether 10 students are chosen randomly from each class (year 4 and year 5). Each student of the class is given an identification number. All of these same identification numbers (written each in a small piece of paper) are also put in a box. The class teacher chooses 10 numbers one by one from the box. Thus 10 students from each class are chosen by lottery. The questionnaires are distributed to the students and recollected by the class teacher after seven days and then are passed back to the researcher for further analysis. Finally, data from 318 students are analysed due to either missing information or received after due date. The missing information is mostly about parents’ education level and income due to their parents’ unwillingness to disclose their personal information. Sample size is calculated using the formula (1/√*N*, where *N* is the number of participants or sample size) to justify the power which shows the significance level at 0.05 up to two decimal places (1//√318 = 0.05). It means, there is only 5% chance of differing the result for sampling error from the actual population (Niles, 2006).

### Research instruments

In this study, a survey questionnaire is administered by the researcher. The questionnaire is used to gather the quantitative data from the participants. The researcher has considered the survey questions to be sufficient to provide data regarding dietary habits. The questionnaires are designed to gather only answers that can easily be quantified and analysed through data analysis procedures. All of the questions are written in Malay language.

#### Questionnaires

The survey question begins with simple questions asking their personal preferences from a list of food and snacks and their consumption frequency. The students are also asked regarding nutrition and healthy eating as well as making food choices. In total there are 14 open-ended and objective questions with options for students.

#### Validity and reliability

In order to make the research findings valid and reliable, critical measures are taken. Any methods for collecting data is selected, procedure should always be examined critically to assess to what extent it is likely to be reliable and valid (Bell, 2005). Reliability ‘refers to whether scores of items on an instrument are internally consistent, stable over time, and whether there was consistency in test administration and scoring’ (Creswell, 2009). In order to enhance the reliability and reduce ambiguity of the questionnaire items, experts’ opinions have been taken and the questionnaire is corrected based on their suggestions to improve the validity. Validity tells us whether an item or instrument measures or describes what it is supposed to measure or describe (Bell, 2005).

The adapted questionnaire is obtained from the Hong Kong Baseline study (Department of Health, 2009). As Malaysia has a different culture, a simple pilot study is conducted to test the validity and reliability of the questions. A total of 30 students are randomly selected and the survey questions are distributed to them. It is noted that these respondents are excluded from the final study. This pilot study shows that majority of the questions is valid and has produced reliable results for analysis though few items are modified. The Cronbach’s Alpha of the instrument is reported in between 0.60 and 0.90.

### Ethical considerations

The questionnaires are assured to contain no prejudice, racial stigmatization, demeaning nature, cynical notion and sarcasm or anything provocative, questions that instigate, or implying of incompetent parenting, mismanaged health and nutrition of students and so forth. The questions are thoroughly screened and rephrased to avoid misunderstanding of the intention of the questions. The questions are also written in a very objective manner, simple and straightforward, clear and specific. Aside from that, the researcher also respects the school’s privacy and decisions during the survey. It is pertinent that the researcher comply with the rules given by the school to ensure smooth response from the participants. The permission is taken from University of Malaya Research ethic Committee before conducting the survey (UM.TNC2/RC/H&E/UMREC-45).

### Data collection procedure

The researchers have performed the survey by approaching individually the schools on Sundays (working day in Kulim, different than other part of Malaysia) during schooling hours, starting from 8 a.m. until 3 p.m. basically; the work flow of the researcher is simplified in the following arrangements:

1. Enter school (walk-in).
2. Request permission with an official letter.
3. Pass the survey questions for students and parents to the administrative staff.
4. Leave the school.
5. Collect survey questions after a week.

The procedure in collecting data is simple and direct and does not require waiting for approval. Schools that refused to cooperate are not included in the study. An immediate approval from supportive schools is highly appreciated and expedited the data collection process.

### Data analysis

There are two main types of statistics used in this study: descriptive and inferential statistics.

#### Descriptive statistics

Raw data obtained from the survey is presented in a form of percentages, frequency and standard deviations. Descriptive statistics is used for all data representation of all survey questions. Generally, descriptive statistics is the easiest form of summarizing data in a presentable format. This simple statistics is performed in Statistical Package for Social Science version 20.0. In identifying knowledge about healthy food, six pairs of food are given in the questionnaire. Students are asked to choose the healthiest food among the six pairs. Students are asked to choose the food choices which they prefer among the six pairs. Each pair contains one healthy and another unhealthy food in accordance with the healthy food guide. Data are analysed describing frequency and percentage for each pair. In identifying students’ attitude to their preferred food, the same six pairs of food and drinks are given in the questionnaire. Data are also analysed describing frequency and percentage for each pair. For identifying food practices, students’ are required to identify their daily dietary habit in the week prior to the questionnaire being carried out. Based on their last seven days practices, data are analysed using frequency and percentage.

#### Inferential statistics

Inferential statistics are used to analyse the data obtained for research question number 2, 3 and 4. Independent sample *t*-test is used to analyse the difference of the respondents’ knowledge, attitude and practices in terms of their gender. One-way ANOVA is used to analyse the difference of the respondents’ knowledge, attitude and practices in terms of parents’ income and education level.

### Demographic information of students

The demographic information of the students is presented in Table 1, which shows the year group, gender, ethnicity, parents’ academic background and income. The data shows that the Year 5 students are found to be the highest number of respondents in this research, which is 186 of them (58.5%) as compared to the Year 4 students who are only 132 (41.5%). As usual, the highest respondents are the female student which is 171 of them (53.8%) compared to the male students who are only 147 (46.2%). The data also shows that the respondents are from three ethnicities. The highest ethnicity which the respondents gathered is Malay with 230 (72.3%) and followed by Indian with 67 respondents (21.1%). The Chinese respondents are the least with only 21 of them (6.6%). The parents’ academic background item is categorized into four categories which are primary school, secondary school, matriculation and college or higher learning institution. The data shows that highest is secondary school with 156 respondents (49.1%), followed by college or higher learning institution, 120 respondents (37.7%) and primary school with 34 respondents (10.7%). The least is parents with matriculation academic background, eight of them (2.5%). The highest respondents for family income less than RM3999 are 182 (57.2%), followed by RM4000-RM7999 with 100 (31.4%) and range from RM8000-RM9999 has 21 respondents (6.6%). The least respondents (15) are from family income of RM10000 and above (4.7%).

**Table 1:** Demographic information of students (*N* = 318).

Student Background	Number	Percentage
**Year**		
4	132	41.5
5	186	58.5
**Gender**		
Male	147	46.2
Female	171	53.8
**Race**		
Malay	230	72.3
Chinese	21	6.6
Indian	67	21.1
**Parents’ Academic Background**		
Primary School	34	10.7
Secondary School	156	49.1
Matriculation	8	2.5
College/Higher Learning Institution	120	37.7
**Family Income**		
Less than RM3999	182	57.2
*RM4000–RM7999	100	31.4
RM8000–RM9999	21	6.6
RM10000 and above	15	4.7

**Note:**

* Malaysian Ringgit.

### Knowledge, attitudes and practices of school students on healthy eating habit

Knowledge, attitudes and the practices of primary school students on healthy eating is presented below.

### Knowledge of school students on healthy eating habit

In identifying knowledge about healthy food, six pairs of food are given in the questionnaire. Students are asked to choose the healthiest food among the six pairs. Based on the Table 2, it shows that the student are able to identify the healthy food choices. The food choice which has the highest rate are the correct choice of healthy foods like soya sauce drumstick with 270 respondents (84.9%), orange juice with 315 respondents (99.1%), raisin bread with 304 respondents (95.6%), yoghurt with 286 respondents (89.9%) and spaghetti with fresh tomato sauce with 280 respondents (88.1%). The last choice has a different result as 280 respondents (88.1%) who have selected fried noodles with chicken as healthy food instead the fried noodles with vegetables which has only 38 respondents (11.9%). This shows that the students are a little confused and believe that chicken is much healthier compared to vegetables.

**Table 2:** Knowledge of healthier food choices of 318 students aged 10–11 years in standard 4–5 in Kulim district, Malaysia.

Food/Drinks						
Item		Freq	%	Item	Freq	%
1	Deep fried drumstick	48	15.1	Soya sauce drumstick	270	84.9
2	Carbonated drink	3	0.9	Orange Juice	315	99.1
3	Raisin bread	304	95.6	Hot dog	14	4.4
4	Ice cream	32	10.1	Yoghurt	286	89.9
5	Hamburger + fries	38	11.9	Spaghetti + tomato	280	88.1
6	Fried noodles + *chic	280	88.1	Fried noodles + *vege	38	11.9

**Note:**

* Chicken, Vegetable.

### Students’ food preference attitude

In identifying students’ preference in food, the same six pairs of food and drinks are given in the questionnaire. Students are asked to choose the food choices which they prefer among the six pairs. Based on the Table 3, it shows there is a difference in answers between students’ preference and their perceived opinions on what healthy food should be. The percentages in students’ food preferences are generally lower. For instance, although many students perceive deep fried drumstick, ice-cream and carbonate drink as unhealthy foods, more than half of the respondents still prefer eating these foods despite the fact that they are an unhealthy choice. Food like deep fried drumstick has higher score of 161 respondents (50.6%) while soya sauce drumstick has only 157 respondents (49.4%) and carbonated drink has the higher respondents of 255 (80.2%) while orange juice has only 63 respondents (19.8%). Raisin bread has 210 respondents (66%) which is higher than hotdog which has 108 respondents (34%), yoghurt with 91 respondents (28.6%) while ice cream has higher score of 227 respondents (71.4%) and spaghetti with fresh tomato sauce has 169 respondents (53.1%) while the hamburger and fries scored less with only 149 respondents (46.9%).

**Table 3:** Attitude for healthy food preference of 318 students aged 10–11 years in standard 4–5 in Kulim district, Malaysia.

Food/Drinks						
Item		Freq	%	Item	Freq	%
1	Deep fried drumstick	161	50.6	Soya sauce drumstick	157	49.4
2	Carbonated drink	255	80.2	Orange Juice	63	19.8
3	Raisin bread	210	66	Hot dog	108	34
4	Ice cream	227	71.4	Yoghurt	91	28.6
5	Hamburger + fries	149	46.9	Spaghetti + tomato	169	53.1
6	Fried noodles + *chic	192	60.4	Fried noodles + *vege	126	39.6

**Note:**

* Chicken, Vegetable

### Practices—students’ daily dietary habit

Students’ are required to identify their daily dietary habit in the week prior to the questionnaire being carried out.

According to Table 4, the percentage of students consuming fruits, vegetables and dairy products more than twice are on an average level between 54.4% (173 respondents), 46.2% (147 respondents) and 52.8% (168 respondents). Grains are also consumed more than twice with 68.9% (219 respondents). However, there is a high average of consumption in unhealthy eating such as having deep fried food 34.9% (111 respondents) and drinks with added sugar with 50.6% (161 respondents) once in a week. Foods that are high in sugar, salt and fat are consumed once in a week. Those who do not reach the recommended frequency stand between 52.8% (168 respondents), 41.8% (133 respondents) and 43.7% (139 respondents). From this result, it is understood that the students are having a positive attitude towards their daily dietary habits.

**Table 4:** Practices of healthy food choices of 318 students aged 10–11 years in standard 4–5 in Kulim district, Malaysia.

		Daily									
		More than twice		Twice		Once		Never		Do not know	
		*F*	*%*	*F*	*%*	*F*	*%*	*F*	*%*	*F*	*%*
A	Fruits	173	54.4	58	18.2	61	19.2	11	3.5	15	4.7
B	Vegetables	147	46.2	90	28.3	46	14.5	23	7.2	12	3.8
C	Dairy products (milk, cheese)	114	35.8	56	17.6	97	30.5	39	12.3	12	3.8
D	Meat, chicken, fish	168	52.8	88	27.7	56	17.6	2	0.6	4	1.3
E	Grains (rice, noodle)	219	68.9	67	21.1	28	8.8	4	1.3	–	–
F	Fried and deep fried food (fried chicken, French fries)	86	27	87	27.4	111	34.9	22	6.9	12	3.8
G	Drinks with added sugar (carbonated drink, cordial drinks)	40	12.6	37	11.6	161	50.6	69	21.7	11	3.5
H	Food high in sugar (sweets, chocolate)	41	12.9	47	14.8	168	52.8	48	15.1	14	4.4
I	Food high in salt (preserved fruits)	18	5.7	14	4.4	133	41.8	139	43.7	14	4.4
J	Food high in fat (snacks, ice cream)	50	15.7	73	23	139	43.7	46	14.5	10	3.1

### Differences of knowledge, attitude and practices based on students’ gender

H_O1_: There is no significant difference of knowledge, attitude and practices based on students’ gender.

An independent samples *t*-test (Table 5) is conducted to compare the mean of knowledge, attitude and practices for males and females student. Based on the *p* value for knowledge (*p* = 0.76), attitude (*p* = 0.68) and practices (*p* = 0.50), this study finds that the assumption of *p* < 0.05 is not met. Thus, the null hypothesis H_O1_ is accepted. This finding indicates that there are no significant differences of knowledge, attitude and practices based on student’s gender.

**Table 5:** Independent sample *t*-test for the differences of knowledge, attitude and practices based on students’ gender (*N* = 318).

Construct	Male (*N* = 147)		Female (*N* = 171)		df	*t*-value	*p*
	*Mean*	*S.D*	*Mean*	*S.D*			
Knowledge	9.35	1.08	9.42	1.03	316	0.29	0.76
Attitude	1.91	0.51	1.94	0.55	316	0.41	0.68
Practice	1.21	0.53	1.19	0.49	316	0.68	0.50

### Differences of knowledge, attitude and practices based on parents’ academic backgrounds

H_O2_: There is no significant difference of students’ knowledge, attitude and practices of healthy eating habit based on parents’ academic backgrounds.

A one-way ANOVA between groups analysis of variance (Table 6) is conducted to explore the mean differences of knowledge, attitude and practices based on four group of parent’s academic background. In Table 6, the *F* distribution for knowledge (2.49), attitude (0.42) and practices (1.22) are smaller than the critical *F* value 2.42. Therefore, the H_O2_ cannot be rejected. Based on the *p* value for knowledge (*p* = 0.60), attitude (*p* = 0.74) and practices (*p* = 0.30), this study finds that the assumption of *p* < 0.05 is not met. Thus, the null hypothesis H_O2_ is accepted. This finding indicates that there are no significant differences of knowledge, attitude and practices of students’ eating habit based on four group of parent’s academic background.

**Table 6:** One-way ANOVA for the differences of knowledge, attitude and practices based on parents’ academic background (*N* = 318).

Construct		Sum of squares		df	Mean square	*F*	*p*
Knowledge	Between group	8.14		3	2.71	2.49	0.60
	Within group	341.51		314	1.08		
	Total	349.65		317			
Attitude	Between group	0.36		3	0.12	0.42	0.74
	Within group	90.15		314	0.29		
	Total	90.51		317			
Practice	Between group	0.97		3	0.32	1.22	0.30
	Within group	83.17		314	0.26		
	Total	84.14		317			

### Differences of knowledge, attitude and practices based on parents’ incomes

H_O3_: There is no significant difference of knowledge, attitude and practices of students’ eating habit based on parents’ incomes.

A one-way ANOVA between groups analysis of variance (Table 7) is conducted to explore the mean differences of knowledge, attitude and practices based on four group of parent’s income. Based on the *p* value for knowledge (*p* = 0.015), attitude (*p* = 0.44) and practices (*p* = 0.58), this study finds that only the comparison of knowledge is less than 0.05 (*F* = 3.57 which is higher than critical value 2.42, df (3, 314)); while for the other two constructs *F* value are 0.91 for attitude and 0.66 for practices which are smaller than the critical *F* value of 2.42. Accordingly, the assumption of *p* < 0.05 is not met. It means that the null hypothesis H_O3_ is partially rejected. Result shows, there is a significant difference in mean knowledge for the four income group while there are no differences in mean attitude and practices of building students healthy eating habits in terms of parents’ income differences. The post hoc LSD (Table 8) shows that the significance difference occurs between 8,000 and 9,999 income group and the other three groups.

**Table 7:** One-way ANOVA for the differences in knowledge, attitude and practices based on parents’ income (*N* = 318).

Construct		Sum of squares		df	Mean square	*F*	*p*
Knowledge	Between group	11.52		3	3.84	3.57	0.015
	Within group	338.13		314	1.08		
	Total	349.65		317			
Attitude	Between group	0.78		3	0.26	0.91	0.44
	Within group	89.74		314	0.29		
	Total	90.52		317			
Practice	Between group	0.53		3	0.18	0.66	0.58
	Within group	83.62		314	0.27		
	Total	84.15		317			

**Table 8:** Post hoc LSD for the differences in knowledge based on parents’ income.

Pair	Mean differences	*p*
<= RM3999 & RM8000-RM9999	0.73	0.002
RM4000 – RM7999 & RM8000-RM9999	0.55	0.029
>= RM10000 & RM8000-RM9999	0.84	0.017

## Discussion

### Students’ knowledge of healthier food choice

The study finds most students having a good knowledge about healthy food choices. For example, in the selection of healthy drinks, 315 students (99.1%) have managed to pick orange juice as healthier than carbonated drinks and yoghurt as well as the selection of healthier beverages than ice cream (89.1%). A similar situation identified in the selection of healthy food, where 270 students (84.9%) have chosen the soy sauce drumstick instead of choosing deep fried drumstick. This means that students understand well the definition of healthy food and the types of food that are considered healthy. This finding is aligned to Lin et al. (2008) in Taiwan and Hoque et al. (2016) in Malaysia who have also found that primary school children acquire simple knowledge in nutrition. However, the percentage of students who has answered the test correctly is only at the rate of 67–71%. Among the other factors that influence students’ knowledge about nutrition and healthy eating is the influence of family and the media. Dixey et al. (2001) explains that most children already understand about balanced diet and its relation to health. In addition, the influence of peers has also helped them understand about healthy eating. This shows that most students are generally aware of the necessity of different food groups, the principle of balanced diet, the skill of selecting foods to achieve the balanced, and the comparison of foods in terms of specific nutrients. Children in general value the importance of nutrition, but they are not concern of the health benefit of foods in food selections (Lin et al., 2008). The study reports that students have good knowledge in the pre-test, before the intervention starts. This situation indicates that school children have already existing knowledge about healthy eating. This is aligned with Piperakis et al. (2004) finding which reveals students’ understanding on the relationships between healthy eating and physical and mental development.

### Knowledge, attitude and practices based on students’ gender

The study finds no significant difference of knowledge on students’ healthy eating habit based on students’ gender. This finding contradicts with Naeeni et al. (2014) and Skardal et al. (2014) who have found that there is a tendency for girls to have a healthier diet than boys, with greater intake of fruits and vegetables (girls intake in median 3.5 units per day and boys 2.9 units per day), and lower intake of soft drinks (girls 0.25 L in median versus boys 0.5 L per week). But Lin et al. (2008) have found no statistically significant differences between genders for nutrition knowledge.

The study also finds no significant difference of attitude and practices of students’ healthy eating habit based on students’ gender. This result is again congruent to Lin et al. (2008), with no statistically significant differences between genders for nutrition attitude and practices. But Petrauskienė, Žaltauskė & Albavičiūtė (2015) have contrast result of significant differences in terms of attitude and practices in breakfast consumption; girls eat breakfast less frequently than boys.

Even the study has found no significant differences by gender, yet a study shows that there are no gender differences in number of foods tried, liked or disliked, although the significant age-by-gender interaction in number of foods disliked suggests that boys may be more ‘picky’ early, where girls are more so during adolescence (Cooke & Wardle, 2005). Another study reports that girls appear to have healthier eating patterns than boys. This is most evident in their consumption of fruit and vegetables (Bradshaw & Benton, 2012).

### Knowledge, attitude and practices based on parents’ academic backgrounds

The study reports no significant difference between knowledge, attitude and practices of building healthy eating habit in terms of parents’ academic backgrounds. This fact is verified in other research (Manios et al., 1999). The child’s broader social environment, including that of the school (Kennedy, Meyers & Layden, 1996) and the family (Mandell, 1993; Brown & Ogden, 2004) is recognized as an important influence in supporting children’s healthy eating behaviour.

There is found no significant difference based on parents’ academic backgrounds. However, children of mothers with a high educational level consume more pieces of fruit per day, more grams of vegetables per day and are more likely to have breakfast on a daily basis than children of mothers with a low educational level. These findings are in line with van Ansem et al. (2014) who have figured out that parental breakfast consumption mediated the association between maternal education level and children’s breakfast consumption. A contradict result shows that parental educational level and parental unemployment are unrelated to adolescents and children’s breakfast consumption (Pearson, Biddle & Gorely, 2009). A recent study (Vollmer & Baietto, 2017) has found negative association of parental control and children food consumption.

### Knowledge, attitude and practices based on parents’ incomes

The study has revealed a significant difference of knowledge of students’ healthy eating habit based on parents’ incomes. This result is slightly congruent to Venezuelan study (Moya De Sofontes & Dehollain, 1986) of affecting children knowledge by family’s shopping pattern and a tight family budget (Ling, Robbins & Hines-Martin, 2016). Another study shows that parents with higher socio-economic status have reported a significantly better knowledge of dietary guidelines compared to those with lower socio-economic status (Skardal et al., 2014). Though, in this study, no significant difference was found in students’ attitude toward healthy eating habit in relation to parents’ incomes, Nelson (2000) has a totally different finding which explains that healthy foods are frequently more expensive, families in poverty live options or families on lower incomes differ in terms of their education and food culture, leading them to make less healthy food choices in areas with lower availability of healthy food. The result related to students healthy food practices of this study also contradict to some other studies (Skardal et al., 2014; Roos et al., 1999) as they have reported that students from families with higher socio-economic status have reported a significant higher intake of vegetables and fish, and lower intake of soft drinks and fast food than those from lower social economic status. It is evident that there is a strong link between the brands children eat and what their family can afford (Ludvigsen & Sharma, 2004) and has a direct association with children’s academic achievement, health status and parents’ income (Abdollahi et al., 2008; Opoola, Adebisi & Ibegbu, 2016). Evidence shows that parents have expressed a desire for nutrition classes including whole family in which topic areas of interest included guidelines of purchasing and cooking healthier foods, knowing the ways to encourage their children to eat healthier foods and knowing the ways of food labels (Prelip et al., 2011).

As a conclusion, parents’ income levels significantly influence students’ knowledge of building healthy eating habits as parents usually buy the foods based on their monetary capability rather to healthy food knowledge. If parents keep healthy food available at home and model healthy eating in front of them then it influences children’s eating habit (Vollmer & Baietto, 2017).

### Limitations and delimitations

Limitations of the study include the respondents, time, location of survey and self-reported questionnaire. Since the study is performed in Kulim district, the data obtained may not be representative of the students in Kedah state (in which the Kulim district is situated) much less to whole Malaysia. Kulim district is also known to be an area populated mostly by medium income families. Data obtained would generally be influenced by the income status and may show similar tendencies. The students who have participated in the survey are only 10 and 11 years old. They are instructed to take the questionnaire at home and collect parents’ income and education information. It is not sure whether they have taken any other information out of instruction. Furthermore, the survey may produce some obstacles in obtaining data at the same time since more than 10 schools are involved. The study is delimited by the questions that are possible for questioning by comparing to the status of the respondents. Deeper excavation of other pertinent information that influences dietary pattern may not be possible through a survey such as food tolerance, clinical issues, and family tradition and so forth. A mixed method study could be more effective for in depth understanding of the problem.

## Concluding Remarks

The main purpose of the study is to find the relationships between parents education level and income and building of students’ healthy eating habits. In looking into these aspects, it has been found that most students have good knowledge on healthy eating (Table 2) but the frequency distribution (Table 4) of their attitude and practices towards healthy food choices are not in line with their knowledge. The study result indicates that parents’ income is the big factor to influence students’ knowledge as they usually consume and experience the foods within their parents’ income capacity. Thus, there is a gap between students’ knowledge and food practices as parents are the key to model healthy food practices at home. Further study in the areas of parental knowledge on healthy food, parental attitude to build children’s healthy food habit and intention of keeping healthy food at home are highly recommended to get the scenario what students experience at home.

## Supplemental Information

10.7717/peerj.4563/supp-1 Supplemental Information 1 Questionnaire.

10.7717/peerj.4563/supp-2 Supplemental Information 2 Raw data.

## Additional Information and Declarations

### Data Availability

The following information was supplied regarding data availability: The raw data has been supplied as Supplemental Dataset Files.
