# Supplementary material for: Relationships between parents’ academic backgrounds and incomes and building students’ healthy eating habits
Source: PeerJ. 2018 May 3;6:e4563. doi: 10.7717/peerj.4563 (PMC5936631; doi:10.7717/peerj.4563)
Supplement: Supplemental Information 1 [file peerj-06-4563-s001.pdf]

Questionnaires

Survey on Diet of Students
Survey Pemakanan Pelajar

Hello students!
We would like to know about your daily eating habits. Please answer the following questions. Don't get influenced by the others when completing the questionnaire. Please be assured that this is an anonymous questionnaire, only researchers can read the questionnaire and the information given by you will be used for data analysis only.

Apa khabar para pelajar!
Kami ingin mengetahui tentang tatacara pemakanan harian anda. Sila jawab soalan berikut dengan jujur. Jawapan untuk kajian ini dirahsiakan hanya penyelidik yang boleh membaca kaji selidik ini dan segala maklumat yang diberi bertujuan untuk analisa data sahaja.

Please tick (√) the box of your choices
Sila tik (√) pada kotak pilihan anda

Year *Darjah* ☐ 4 ☐ 5

Class number *Kedudukan kelas dalam darjah* ☐

Sex *Jantina* ☐ **Male** *Lelaki* ☐ **Female** *Perempuan*

Kaum *Race* ☐ **Melayu** *Malay* ☐ **Cina** *Chinese* ☐ **India** *Indian*

Date of birth *Tarikh lahir* ☐ **Day** *Hari* ☐ **Month** *Bulan*

Your parents (father/mother) education level :
Tahap pendidikan anda:

○**Primary education or below** *Sekolah rendah*
○**Secondary education (Secondary 1-5)** *Sekolah menengah (Menengah 1-5)*
○**Matriculation** *Matrikulasi*
○**College or above** *Kolej atau pendidikan tinggi*

Monthly household income:
Pendapatan isirumah bulanan:

○**RM 3,999 or less** *RM3,999 atau kurang*
○**RM4,000 – RM7,999** *RM4,000 – RM7,999*
○**RM8,000 – RM9,999** *RM8,000 – RM9,999*
○**RM10,000 and above**

In each of the following pairs, please tick (√) next to the food or drink that you prefer.
Untuk setiap pasangan, sila tik (√) di sebelah makanan atau minuman yang anda lebih gemari.

Knowledge

1. **Which item do you think is the healthier choice in each of the following pairs? Tick (√) next to the food or drink that <u>you think is healthier</u>.**
Antara pasangan berikut, yang manakah merupakan makanan pilihan yang sihat. Tik (√) bagi *makanan atau minuman yang* ***<u>anda rasa lebih sihat</u>***.

A	**Deep-fried drumstick**<br>*Drumstik goreng* 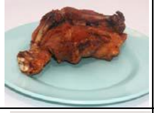		**Soya sauce drumstick**<br>*Drumstik masak kicap* 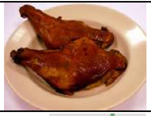	
B	**Carbonated drinks**<br>*Minuman bergas* 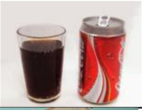		**Pure orange juice**<br>*Jus oren asli* 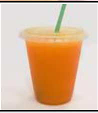	
C	**Raisin whole-meal bread**<br>*Roti serat kismis* 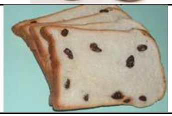		**Hot dog**<br>*Hot dog* 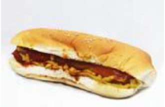	
D	**Ice cream**<br>*Ais krim* 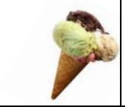		**Yoghurt**<br>*Yoghurt* 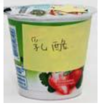	
E	**Spaghetti with fresh tomato sauce**<br>*Spagetti dengan sos tomato* 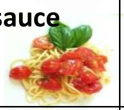		**Hamburger and fries**<br>*Burger dan kentang goreng* 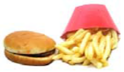	
F	**Fried noodle with vegetables**<br>*Mee goreng dengan sayur* 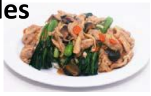		**Fried noodles with chicken**<br>*Mee goreng dengan ayam* 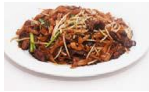	

Attitude

2. **Which item do you prefer of the following pairs? Tick (√) next to the food or drink that <u>you prefer</u>.**
Item manakah yang anda lebih suka daripada pasangan berikut? Tandakan (√) di sebelah makanan atau ***minuman yang anda suka****.*

A	**Deep-fried drumstick**<br>*Drumstik goreng* 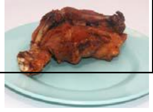		**Soya sauce drumstick**<br>*Drumstik masak kicap* 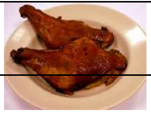	

B	**Carbonated drinks**<br>*Minuman bergas* 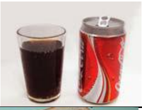		**Pure orange juice**<br>*Jus oren asli* 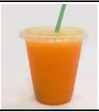	
C	**Raisin whole-meal bread**<br>*Roti serat kismis* 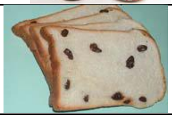		**Hot dog**<br>*Hot dog* 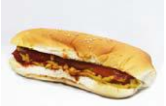	
D	**Ice cream**<br>*Ais krim* 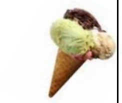		**Yoghurt**<br>*Yoghurt* 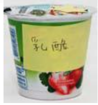	
E	**Spaghetti with fresh tomato sauce**<br>*Spagetti dengan sos tomato* 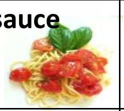		**Hamburger and fries**<br>*Burger dan kentang goreng* 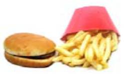	
F	**Fried noodle with vegetables**<br>*Mee goreng dengan sayur* 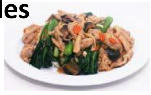		**Fried noodles with chicken**<br>*Mee goreng dengan ayam* 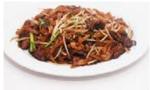	

Practices

3. **In the past one week, the number of times I have eaten the following food <u>on average every day</u> is about:**
Pada minggu lepas, kekerapan pengambilan makanan berikut ***<u>secara purata setiap hari</u>*** *adalah:*

		**Daily**<br>*Kekerapan*				
		**More than twice**<br>*Lebih dua kali*	**Twice**<br>*Dua kali*	**Once**<br>*Sekali*	**Never**<br>*Tidak pernah*	**Do not know**<br>*Tidak tahu*
A	**Fruits**<br>*Buah*					
B	**Vegetables**<br>*Sayur*					
C	**Dairy products (milk, cheese)**<br>*Produk tenusu (susu, keju)*					
D	**Meat, chicken, fish**<br>*Daging, ayam, ikan*					
E	**Grains (rice, noodle)**<br>*Bijirin (nasi, mee)*					
F	**Fried and deep fried food (fried chicken, French fries)**<br>*Makanan yang digoreng (ayam goring, kentang goreng)*					
G	**Drinks with added sugar (carbonated drink, cordial drinks)**					

	Minuman yang ditambah gula (air bergas, air kodial)					
H	**Food high in sugar (sweets, chocolate)**<br>*Makanan yang mengandungi kandungan gula yang tinggi (gula-gula, coklat)*					
I	**Food high in salt (preserved fruits)**<br>*Makanan yang mengandungi kandungan garam yang tinggi (jeruk buah)*					
J	**Food high in fat (snacks, ice cream)**<br>*Makanan yang mengandungi lemak yang tinggi (makanan ringan, ice cream)*					

End of questionnaire. Thank you!
